# Supplementary material for: Wolbachia Natural Infection of Mosquitoes in French Guiana: Prevalence, Distribution, and Genotyping
Source: Microorganisms. 2024 Sep 30;12(10):1994. doi: 10.3390/microorganisms12101994 (PMC11509720; doi:10.3390/microorganisms12101994)
Supplement: Supplementary file 1 [file microorganisms-12-01994-s001.zip › microorganisms-3190665-supplementary.pdf]

Wolbachia Natural Infection of Mosquitoes in French Guiana: Prevalence, Distribution, and Genotyping

Emmanuelle Clervil [1], Amandine Guidez [1], Stanislas Talaga [1], Romuald Carinci [1], Pascal Gaborit [1], Anne Lavergne [2], Sourakhata Tirera [2] and Jean-Bernard Duchemin [1,*]

[1] Unité d'Entomologie Médicale, Institut Pasteur de la Guyane, Cayenne 97300, French Guiana
[2] Laboratoire d'Interaction Hôte-Virus, Institut Pasteur de la Guyane, Cayenne 97300, French Guiana
* Correspondence: jean-bernard.duchemin@pasteur.fr

SUPPLEMENTAL MATERIAL

Supplemental Table S1. Infection rate by *Wolbachia* bacteria (detected with 16S rRNA RT-qPCR – and WSP PCR [33,36,43] among 45 mosquito species collected in French Guiana. The prevalence of *Wolbachia* is based on the number of positive PCR for the WSP gene.

Species	Positive 16S rRNA RT-qPCR	Prevalence of *Wolbachia* (%) based on RT-qPCR (n tested)	Positive PCR for WSP gene	Prevalence of *Wolbachia* (%) based on PCR (n tested)
Aedeomyia squamipennis	**15**	**100 (15)**	**13**	**86.7 (15)**
Aedes aegypti	**0**	**0 (100)**	**0**	**0 (100)**
Aedes argyrothorax	**0**	**0 (1)**	**0**	**0 (1)**
Aedes fluviatilis	**0**	**0 (12)**	**0**	**0 (12)**
Aedes fulvithorax	**0**	**0 (7)**	**0**	**0 (7)**
Aedes scapularis	**2**	**11 (19)**	**1**	**5.3 (19)**
Aedes serratus	**10**	**48 (21)**	**4**	**19 (21)**
Aedes taeniorhynchus	**0**	**0 (10)**	**0**	**0 (10)**
Anopheles aquasalis	**0**	**0 (9)**	**0**	**0 (9)**
Anopheles darlingi	**3**	**15 (20)**	**0**	**0 (20)**
Anopheles medialis	**1**	**9 (11)**	**0**	**0 (11)**
Anopheles nuneztovari	**0**	**0 (10)**	**0**	**0 (10)**
Coquillettidia albicosta	**8**	**89 (9)**	**8**	**88.9 (9)**
Coquillettidia lynchi	**3**	**27 (11)**	**1**	**9.1 (11)**
Coquillettidia venezuelensis	**16**	**84 (19)**	**12**	**63.2 (19)**

Coquillettidia sp	1	17 (6)	0	0 (6)
Culex coronator	3	30 (10)	1	10 (10)
Culex dunni	2	20 (10)	1	10 (10)
Culex eastor	5	100 (5)	5	100 (5)
Culex idottus	3	75 (4)	3	75 (4)
Culex nigripalpus	0	0 (9)	0	0 (9)
Culex ocellatus	4	40 (10)	4	40 (10)
Culex pedroi	2	67 (3)	2	66.7 (3)
Culex portesi	8	80 (10)	8	80 (10)
Culex quinquefasciatus	10	100 (10)	10	100 (10)
Culex rabanicolus	4	80 (5)	4	80 (5)
Deinocerites magnus	0	0(3)	0	0 (3)
Haemagogus janthinomys	0	0 (3)	0	0 (3)
Limatus durhamii	4	27 (15)	4	26.7 (15)
Uranotaenia calosomata	0	0 (2)	0	0 (2)
Uranotaenia hystera	2	20 (10)	0	0 (10)
Uranotaenia leucoptera	5	50 (10)	5	50 (10)
Uranotaenia lowii	1	100 (1)	1	100 (1)
Mansonia titillans	10	40 (25)	8	32 (25)
Mansonia pseudotitillans	1	50 (2)	1	50 (2)
Psorophora albipes	0	0 (3)	0	0 (3)
Psorophora cingulata	0	0 (2)	0	0 (2)
Psorophora ferox	1	6 (18)	1	5.6 (18)
Psorophora lutzii	2	100 (2)	0	0 (2)
Sabethes quasicyaneus	6	67 (9)	6	66.7 (9)
Toxorhynchites haemorrhoidalis	0	0 (3)	0	0 (3)
Trichoprosopon digitatum	3	75 (4)	3	75 (4)
Wyeomyia luteoventralis	8	40 (20)	5	25 (20)
Wyeomyia occulta	0	0 (8)	0	0 (8)

Table S2. WSP gene sequences and their accession numbers.

WSP sequence accession numbers	WSP sequence accession numbers
BankIt2837621 EC010 PP887524	BankIt2837621 EC348 PP887554
BankIt2837621 EC011 PP887525	BankIt2837621 EC351 PP887555
BankIt2837621 EC134 PP887526	BankIt2837621 EC036 PP887556
BankIt2837621 EC140 PP887527	BankIt2837621 EC363 PP887557
BankIt2837621 EC141 PP887528	BankIt2837621 EC037 PP887558
BankIt2837621 EC142 PP887529	BankIt2837621 EC039 PP887559
BankIt2837621 EC002 PP887530	BankIt2837621 EC393 PP887560
BankIt2837621 EC206 PP887531	BankIt2837621 EC394 PP887561
BankIt2837621 EC207 PP887532	BankIt2837621 EC004 PP887562
BankIt2837621 EC208 PP887533	BankIt2837621 EC043 PP887563
BankIt2837621 EC209 PP887534	BankIt2837621 EC044 PP887564
BankIt2837621 EC233 PP887535	BankIt2837621 EC442 PP887565
BankIt2837621 EC027 PP887536	BankIt2837621 EC443 PP887566
BankIt2837621 EC003 PP887537	BankIt2837621 EC045 PP887567
BankIt2837621 EC300 PP887538	BankIt2837621 EC046 PP887568
BankIt2837621 EC301 PP887539	BankIt2837621 EC047 PP887569
BankIt2837621 EC303 PP887540	BankIt2837621 EC048 PP887570
BankIt2837621 EC305 PP887541	BankIt2837621 EC050 PP887571
BankIt2837621 EC306 PP887542	BankIt2837621 EC052 PP887572
BankIt2837621 EC307 PP887543	BankIt2837621 EC053 PP887573
BankIt2837621 EC308 PP887544	BankIt2837621 EC055 PP887574
BankIt2837621 EC313 PP887545	BankIt2837621 EC0651 PP887575
BankIt2837621 EC315 PP887546	BankIt2837621 EC0652 PP887576
BankIt2837621 EC314 PP887547	BankIt2837621 EC0661 PP887577
BankIt2837621 EC316 PP887548	BankIt2837621 EC0662 PP887578
BankIt2837621 EC212 PP887549	BankIt2837621 EC0671 PP887579
BankIt2837621 EC320 PP887550	BankIt2837621 EC0672 PP887580
BankIt2837621 EC317 PP887551	BankIt2837621 EC007 PP887581
BankIt2837621 EC319 PP887552	BankIt2837621 EC070 PP887582
BankIt2837621 EC344 PP887553	BankIt2837621 EC009 PP887583

Table S3. 16SrRNA gene sequences and their accession numbers.

16SrRNA sequence accession numbers	16SrRNA sequence accession numbers
SUB14510045 EC002 PP905555	SUB14510045 EC301 PP905575
SUB14510045 EC002 PP905555	SUB14510045 EC302 PP905576
SUB14510045 EC007 PP905557	SUB14510045 EC303 PP905577
SUB14510045 EC009 PP905558	SUB14510045 EC305 PP905578
SUB14510045 EC010 PP905559	SUB14510045 EC306 PP905579
SUB14510045 EC011 PP905560	SUB14510045 EC307 PP905580
SUB14510045 EC036 PP905561	SUB14510045 EC308 PP905581
SUB14510045 EC044 PP905562	SUB14510045 EC311 PP905582
SUB14510045 EC041 PP905563	SUB14510045 EC312 PP905583
SUB14510045 EC043 PP905564	SUB14510045 EC316 PP905584
SUB14510045 EC048 PP905565	SUB14510045 EC320 PP905585
SUB14510045 EC050 PP905566	SUB14510045 EC315 PP905586
SUB14510045 EC052 PP905567	SUB14510045 EC317 PP905587
SUB14510045 EC053 PP905568	SUB14510045 EC319 PP905588
SUB14510045 EC055 PP905569	SUB14510045 EC313 PP905589
SUB14510045 EC061 PP905570	SUB14510045 EC347 PP905590
SUB14510045 EC064 PP905571	SUB14510045 EC442 PP905591
SUB14510045 EC0661 PP905572	SUB14510045 EC394 PP905592
SUB14510045 EC070 PP905573	SUB14510045 EC140 PP905593
SUB14510045 EC233 PP905574	
